# Prediction Equation for Lower Limbs Lean Soft Tissue in Circumpubertal Boys Using Anthropometry and Biological Maturation

**DOI:** 10.1371/journal.pone.0107219

**Published:** 2014-09-17

**Authors:** João Valente-dos-Santos, Manuel J. Coelho-e-Silva, Aristides M. Machado-Rodrigues, Marije T. Elferink-Gemser, Robert M. Malina, Édio L. Petroski, Cláudia S. Minderico, Analiza M. Silva, Fátima Baptista, Luís B. Sardinha

**Affiliations:** 1 Faculty of Sport Sciences and Physical Education, University of Coimbra, Coimbra, Portugal; 2 Center for Human Movement Sciences, University Medical Center Groningen, University of Groningen, Groningen, The Netherlands; 3 Institute for Studies in Sports and Exercise, HAN University of Applied Sciences, Nijmegen, The Netherlands; 4 Department of Kinesiology and Health Education, University of Texas at Austin, Austin, Texas, United States of America; 5 Department of Kinesiology, Tarleton State University, Stephenville, Texas, United States of America; 6 Research Centre for Kinanthropometry and Human Performance, Federal University of Santa Catarina, Florianópolis, Santa Catarina, Brazil; 7 Exercise and Health Laboratory, CIPER, Faculdade de Motricidade Humana, Universidade de Lisboa, Cruz-Quebrada, Portugal; Rush University Medical Center, United States of America

## Abstract

Lean soft tissue (LST), a surrogate of skeletal muscle mass, is largely limited to appendicular body regions. Simple and accurate methods to estimate lower limbs LST are often used in attempts to partition out the influence of body size on performance outputs. The aim of the current study was to develop and cross-validate a new model to predict lower limbs LST in boys aged 10–13 years, using dual-energy X-ray absorptiometry (DXA) as the reference method. Total body and segmental (lower limbs) composition were assessed with a Hologic Explorer-W QDR DXA scanner in a cross-sectional sample of 75 Portuguese boys (144.8±6.4 cm; 40.2±9.0 kg). Skinfolds were measured at the anterior and posterior mid-thigh, and medial calf. Circumferences were measured at the proximal, mid and distal thigh. Leg length was estimated as stature minus sitting height. Current stature expressed as a percentage of attained predicted mature stature (PMS) was used as an estimate of biological maturity status. Backward proportional allometric models were used to identify the model with the best statistical fit: ln (lower limbs LST)  = 0.838× ln (body mass) +0.476× ln (leg length) – 0.135× ln (mid-thigh circumference) – 0.053× ln (anterior mid-thigh skinfold) – 0.098× ln (medial calf skinfold) – 2.680+0.010× (percentage of attained PMS) (*R* = 0.95). The obtained equation was cross-validated using the predicted residuals sum of squares statistics (PRESS) method (*R* ^2^_PRESS_ = 0.90). Deming repression analysis between predicted and current lower limbs LST showed a standard error of estimation of 0.52 kg (95% limits of agreement: 0.77 to −1.27 kg). The new model accurately predicts lower limbs LST in circumpubertal boys.

## Introduction

Skeletal muscle mass in the lower extremities is well correlated with a variety of fitness measures in pediatric physiology and health and exercise research [1]–[6]. Oxygen uptake is probably the most frequently reported physical capacity measurement in children and, for example, is not clear what the pattern of change with age or maturation is once values are dimensionally scaled [7], [8]. In heterogeneous samples of boys and men, it has been demonstrated that estimates of lower leg muscle [3] or thigh muscle [6] volume are more relevant allometric scaling denominators for partitioning out the influence of body size on peak oxygen uptake than either body mass or fat-free mass.

Early dissection studies have indicated that the muscles in the lower extremities increase their relative contribution to total muscle mass from about 40% at birth to 55% at maturity [9]. Other studies of regional variation in skeletal muscle were based on radiography and limited largely to the arm and calf [10]. Boys gain about 30% in calf muscle mass width during adolescence [10], but more recent imaging techniques have not yet been used systematically to address this issue in youth.

Multi-scan magnetic resonance imaging (MRI) and computerized axial tomography (CT) are considered the “criterion” methods for evaluating skeletal muscle mass [11]. MRI is not widely available for use in research and clinical practice while CT includes exposure to radiation which limits its application in children and adolescents [12]. Other approaches to estimate skeletal muscle mass, include total-body potassium [13] and bioelectrical impedance [14]. Densitometric techniques rely on specific assumptions concerning the density of fat-free mass (1.1 g/mL) which is assumed to be stable in adults but may vary considerably in children by age, gender, maturity status and perhaps ethnicity [15], [16]. The estimated density of fat-free mass increases from 1.0826 to 1.1013 g/mL in boys from 4 to 23 years [17]. Dual-energy X-ray absorptiometry (DXA) is a non-invasive protocol with a minimal radiation dose (1 mSv or 1/100^th^ of the equivalent radiation exposure of a chest x-ray) [18]. The DXA method provides total and lean soft tissue (LST) estimates of the extremities, and a large portion of total-body skeletal muscle is within the fat-free appendicular compartment [19]. DXA-determined appendicular LST has also been validated against criterion methods such as CT [20], [21].

Though useful, the availability of DXA in field settings is limited. Estimates of LST based on anthropometry are thus potentially useful, especially among youth. A popular anthropometric method [22] characterizes the lower limb as the sum of six truncated cones based on lengths, circumferences and skinfold thicknesses. The method was originally developed among young adults (with 32 male and 15 female) and has been applied in children and adolescents [4], [23], [24]. Anthropometric models for appendicular LST (upper and lower limbs combined) in young athletes of both sexes have also been developed and cross-validated [25]. A recent attempt [26] to cross-validate the regression equation of Jones and Pearson [22] in youth (83 girls, 85 boys, 11.0±0.7 years) noted a significantly higher thigh volume with DXA (4.43±1.23 L) than with the anthropometric estimate (4.39±1.22 L). Addition of body mass and the sum of anterior and posterior thigh skinfolds to the model resulted in a new predictive equation (*R* ^2^ = 0.95). The available research has not systematically considered the potential contribution of biological maturation to inter-individual variability in total and lean soft tissues. Correlations between skeletal age and radiographic widths of upper arm and calf muscle increased from childhood (7–10 years, *r* = 0.16–0.18) to adolescence (11–16 years, *r* = 0.51–0.61) in boys; corresponding correlations in girls, on the other hand, increased with age for arm musculature (7–8 years, *r* = 0.27; 9–14 years, *r* = 0.45) but not for calf musculature (7–8 years, *r* = 0.41; 9–14 years, *r* = 0.39) [27]. The direction of correlations between skeletal age and arm and calf muscle widths in boys across chronological age (CA) groups suggests a need to consider inter-individual differences in maturity status in estimates of lower limbs LST in circumpubertal children. Allowing for the limited data on youth, the present study attempted to develop a non-invasive estimate of lower limbs LST in circumpubertal boys and to cross-validate the new model using DXA measures as the reference.

## Materials and Methods

### Ethics Statement

The study was approved by the Portuguese Foundation for Science and Technology [PTDC/DTP-DES/1178/2012] and by the scientific board of the Faculty of Human Kinetics at the Technical University of Lisbon. The study was conducted in accordance with the Declaration of Helsinki for human studies by the World Medical Association [28]. All participants, parents or legal guardians were informed about the objectives of the study and provided appropriate informed assent and written informed consent.

### Participants

The present study is limited to healthy boys of European ancestry (*n* = 75), 10–13 years of age. Participants were recruited voluntarily from the school population in the Lisbon metropolitan area (Portugal).

### Chronological Age and Anthropometry

Chronological age was calculated as the difference between date of birth and date of measurement. Anthropometry was performed by a single experienced observer following standard procedures [29]. Stature and sitting height were measured to the nearest 0.1 cm with a Harpenden stadiometer (model 98.603, Holtain Ltd, Crosswell, UK and Harpenden sitting height table, model 98.607, Holtain Ltd, Crosswell, UK, respectively) and body mass was measured to the nearest 0.1 kg using an electronic scale (Seca, Hamburg, Germany). Leg length was estimated as stature minus sitting height. Circumference at the gluteal furrow (highest possible horizontal circumference), mid-thigh (largest mid-thigh circumference) and distal thigh (minimum circumference above the knee) were measured on the right site of the body. Lengths between each circumference level were also measured to estimate total thigh length. Anterior and posterior mid-thigh and medial calf skinfolds were measured to the nearest mm using a Lange Caliper (Beta Technology, Ann Arbor, MI, USA). Technical errors of measurement were previously reported for the anthropometric dimensions [26] and all were well within the range of several health surveys in the United States and a variety of field surveys [30]. Mid-thigh circumference (*C*_m-t_) was corrected for subcutaneous adipose tissue thickness [31]. The corrected mid-thigh circumference (*CC*_m-t_) was calculated as *CC*_m-t_ = *C*_m-t_ – πS, where *S* stands for the skinfold measurement, which is assumed to be twice the subcutaneous adipose tissue thickness.

### Biological Maturity

Chronological age, stature, and body mass of each boy and midparent stature were used to predict mature (adult) stature using the Khamis–Roche protocol [32]. Parent heights were extracted from national identification cards which included height measured to the nearest centimeter. Measurements were taken by experienced, but not necessarily trained observers. A similar protocol was used in previous studies [33], [34]. The median error bound (median absolute deviation) between actual and predicted mature stature (PMS) at 18 years of age is 2.2 cm in boys [32]. Ages at attaining specific percentages of mature stature are related to ages at attaining specific skeletal ages and stages of puberty in boys followed longitudinally from late childhood through adolescence [35]. Percentage of attained PMS (% PMS) is also related to skeletal age in youth football (American) and soccer players of the same age range as the study sample [33], [36]. Percentage of attained PMS was expressed as a *z*-score relative to age-specific (half-year intervals) means and standard deviations from the Berkeley Guidance Study [37]; the *z*-score (*z* PMS) was used as the indicator of maturity status for each boy.

### Dual energy X-ray absorptiometry

A Hologic Explorer-W, fan-bean densitometer, software QDR for Windows version 12.4 (Hologic, Waltham, MA, USA) was used to perform whole body scans. The procedure also allows measurement of segmental composition: arm, leg and trunk. Daily calibration of the scanner was performed using a phantom spine containing composites of bone, fat and LST. Participants were positioned on the scanner bed according to manufacturer recommendations. A single lab technician positioned the participants and performed the scans according to the manual provided by the manufacturer. The lower limbs on each image were sectioned as follows: all tissue distal to a line drawn through and perpendicular to the axis of the femoral neck and angled with the pelvic brim to the phalange tips. Total lower limb composition was estimated by adding the mass of the left and right legs. Mean variation between measured and reconstructed absolute whole-body mass with DXA software was 0.9%. Based on test-retest in 10 individuals, the coefficients of variation for percent body fat and LST were 1.6% and 0.8%, respectively.

### Statistical analyses

Visual inspections of data were made using stem-and-leaf diagram and box-and-whisker plots to examine central tendency, variability, distributions and potential outliers. Subsequently, Gaussian distribution of variables was confirmed with normal Q-Q plots, de-trended normal Q-Q plots and Kolmogorov-Smirnov (*K-S*) test with Lilliefors significance correction. Descriptive statistics were calculated for the total sample (mean, standard deviation and range).

A backward multiple linear regression on ln *y*, based on proportional allometric models [4], [38], was used to fit the unknown parameters that best predict lower limb LST determined with DXA. Based on analytical [i.e., Pearson's product moment correlation coefficients (*r_y,x_*) between lower limbs LST_DXA_ (*y*) with CA, maturity status and body descriptors (*x*)] and biological assumptions, the new model (NM) considered CA, CA^2^, % PMS or *z* PMS as exponential terms in addition to body mass, leg length, *C*_m-t_, stature × *CC*_m-t_^2^, anterior and posterior mid-thigh and medial calf skinfolds:
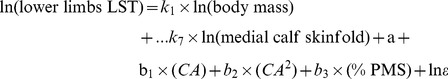
(1)


A tolerance >0.10 and a variance inflation factor <10 was set to avoid collinearity between the explanatory variables [39].

The new model was internally cross-validated by using the predicted residuals sum of squares (PRESS) statistics method [40]. PRESS statistics uses all cases and is considered a statistical jackknife procedure [41]. The technique produces as many models as the number of cases. An independent model is obtained based on *n*-1 data subsets for each participant and this permits the determination of individual residuals. A PRESS residual for a given participant is the difference between the actual response of the left-out case and the predicted response. Prediction of the respective case involves a different prediction equation for a statistical fit that excludes the case [40]. Overall, the PRESS statistic is a function of the sum of squares (SS) of all residuals. The PRESS statistic is always higher than the error (i.e., SS) from MANOVA results. Alternative measures of model adequacy (*R* ^2^_PRESS_) and standard error of estimation (SEE_PRESS_) were also calculated [40].

Agreement between the reference method (lower limbs LST_DXA_) and the estimation (lower limbs LST_NM_) was assessed using Deming regression (i.e., least products regression method which accounts for the error of both *x* and *y* variables) and standard error of estimation (S*_y·x_*) [42]. Data were then visually inspected by plotting residuals (prediction errors) against predicted values. This plot is similar to the plot suggested by Bland and Altman [43], except that predicted values of lower limbs LST (using the new developed equation) are plotted on the *x*-axis rather than the average of predicted and measures of lower limbs LST [25]. To test for proportional bias, Bland and Altman [43] have recommended plotting error against the average of two the methods. Although they have demonstrated that this is correct when considering the agreement of two existing methods, Hopkins [44] has demonstrated that in criterion/reference validity studies this method will indicate positive proportional bias when no proportional bias exists. This is due to the mathematical relation that occurs when a method is calibrated against a reference using least-squares regression; only vertical error (error in predicting the criterion) is minimized around the regression slope [45]. If errors were associated with the magnitude of the values, heteroscedasticity was examined by calculating the correlation coefficients between the absolute differences and the predicted values of lower limbs LST. Constant error variance (homoscedasticity) can be assumed if the correlations approach zero [46], [47]. Correlation coefficients were interpreted as follows: trivial (*r*<0.1), small (0.1<*r*<0.3) moderate (0.3<*r*<0.5), large (0.5<*r*<0.7), very large (0.7<*r*<0.9), nearly perfect (*r*>0.9) and perfect (*r* = 1) [48]. Finally, if measurement differences were normally distributed, reliability was assessed on the original scale using the standard error of measurement and 95% limits of agreement [43].

Statistical analyses were performed using IBM SPSS version 19.0 software (SPSS, Inc., IBM Company; NY, USA), GraphPad Prism version 5.03 software (GraphPad Software, Inc.; La Jolla, CA, USA) and MedCalc version 12.2.1 software (MedCalc; Mariakerke, Belgium). Alpha level was set at 0.05.

## Results

Descriptive statistics for CA, estimated maturity status and DXA body composition are summarized in Table 1. All variables fitted a normal distribution (*K-S* = 0.37 to 1.18; *p*>0.05). Mean percentage of attained PMS (81.5±2.3%) for the present sample was equivalent of that of the sample on which the physical maturity-prediction protocol was based (81.6±1.2% at 11.0 years) [49]. The means and standard deviations for percent of total body fat_DXA_, LST_DXA_, lower limbs fat percentage_DXA_ and lower limbs LST_DXA_ were 26.4 (±8.1%), 29.0 (±4.2 kg), 31.7 (±8.3%) and 10.0 (±1.7 kg), respectively.

**Table 1:** Descriptive statistics (*n* = 75) for chronological age, maturity status and body composition, and results of the Kolmogorov–Smirnov test for checking the normality of the distribution.

	Mean ± SD	Range (min – max)	Kolmogorov–Smirnov	
			Value	*p*
Chronological age (years)	11.12±0.69	10.27 to 13.08	1.18	0.12
Percentage of PMS (%)	81.5±2.3	77.3 to 90.3	0.70	0.72
*z*-score for PMS (*z* PMS)	0.04±0.92	−2.07 to 2.40	0.74	0.65
DXA total body				
BMD (g•cm^−2^)	0.84±0.06	0.69 to 0.96	0.37	0.99
BMC (kg)	1.22±0.17	0.86 to 1.66	0.55	0.93
Fat (%)	26.4±8.1	14.0 to 44.6	0.77	0.60
Fat (kg)	11.2±5.8	3.6 to 27.6	1.03	0.24
Lean soft tissue (kg)	29.0±4.2	20.7 to 39.8	0.73	0.66
DXA lower limbs				
BMD (g•cm^−2^)	0.84±0.07	0.68 to 1.04	0.51	0.96
BMC (kg)	0.44±0.08	0.30 to 0.68	0.87	0.43
Fat (%)	31.7±8.3	16.0 to 50.2	0.56	0.92
Fat (kg)	4.9±2.3	1.3 to 10.9	1.16	0.13
Lean soft tissue (kg)	10.0±1.7	6.9 to 13.9	0.74	0.64

Abbreviations: PMS, predicted mature stature; DXA, dual x-ray absorptiometry; BMD, bone mineral density; BMC, Bone mineral content.

**Figure 1:**
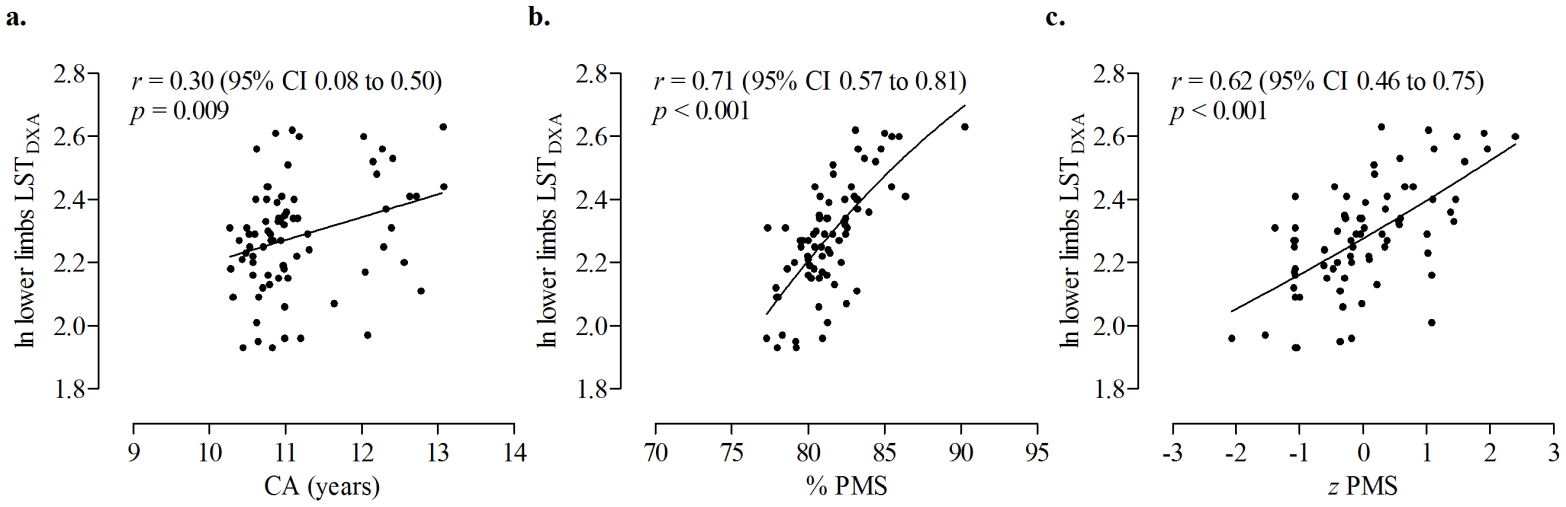
Relationship of the ln transformed lower limbs lean soft tissue (LST_DXA_) with chronological age (CA, panel a), percentage of predicted mature stature attained at the time of study (% PMS, panel b), and *z*-score for % PMS (*z* PMS, panel c).

Relationships between ln lower limbs LST_DXA_ and the chronovariables [i.e., CA, percent of attained PMS and z-score for age (% attained PMS)] are illustrated in Figure 1 (panels a–c). The relationship between CA and lower limbs LST_DXA_ is linear, while relationships between % PMS and *z* PMS with lower limbs LST_DXA_ are somewhat non-linear. The correlation between CA and lower limbs LST_DXA_ is 0.30, while the corresponding correlations for % PMS and *z* PMS are 0.71 and 0.62, respectively (*p*<0.01).

Anthropometric characteristics for the total sample, results of the Kolmogorov–Smirnov test, and bivariate correlations of anthropometry with CA and estimated maturity status are shown in Table 2. The current sample has a mean stature and mean body mass which approximate, respectively, the 56^th^ and 60^th^ age-specific percentiles for U.S. boys [50]. Correlations between CA and maturity status and anthropometric variables ranged from trivial (*r* = 0.01) to very large (*r* = 0.73), suggesting the possibility of collinearity occurrence between some exponential terms and body size descriptors.

**Table 2:** Descriptive statistics (*n* = 75) for anthropometric variables, results of the Kolmogorov–Smirnov test for checking the normality of the distribution, and bivariate correlations of anthropometry with chronological age and maturity status.

	Mean ± SD	Range (min – max)	Kolmogorov–Smirnov		Correlation					
					CA		% PMS		*z* PMS	
			Value	*p*	*r*	95% CI	*r*	95% CI	*r*	95% CI
Stature (cm)	144.8±6.4	127.9 to 163.5	0.52	0.95	0.36	0.15 to 0.55	0.73	0.60 to 0.82	0.58	0.41 to 0.71
Body mass (kg)	40.2±9.0	24.4 to 66.5	1.17	0.13	0.15	−0.01 to 0.37	0.64	0.48 to 0.76	0.71	0.57 to 0.81
Lengths (cm)										
Leg	69.6±4.2	60.2 to 78.5	0.43	0.99	0.34	0.13 to 0.53	0.63	0.47 to 0.75	0.49	0.30 to 0.65
Thigh	27.8±3.9	17.3 to 36.2	0.58	0.89	0.12	−0.11 to 0.34	0.15	−0.08 to 0.36	0.06	−0.17 to 0.29
Circumferences (cm)										
Proximal thigh	46.8±6.2	36.5 to 66.7	0.97	0.30	0.05	−0.18 to 0.27	0.54	0.35 to 0.68	0.68	0.54 to 0.79
Mid-thigh	41.3±5.4	26.0 to 54.0	0.85	0.47	0.16	−0.08 to 0.37	0.52	0.33 to 0.67	0.54	0.35 to 0.68
Distal thigh	34.3±4.4	27.5 to 47.0	1.19	0.12	0.01	−0.22 to 0.23	0.47	0.27 to 0.63	0.64	0.49 to 0.76
Skinfold thickness (mm)										
Anterior mid-thigh	21±8	7 to 42	0.85	0.47	−0.19	−0.40 to 0.04	0.21	−0.02 to 0.41	0.51	0.32 to 0.66
Posterior mid-thigh	18±8	6 to 37	1.09	0.19	−0.07	−0.30 to 0.16	0.31	0.09 to 0.50	0.53	0.34 to 0.68
Medial calf	14±6	4 to 28	1.11	0.17	−0.04	−0.27 to 0.19	0.41	0.20 to 0.58	0.62	0.46 to 0.74

Abbreviations: CA, Chronological age; PMS, predicted mature stature; *r*, Pearson's product moment correlation coefficient; 95% CI, 95% confidence interval.

The allometric regression model for the prediction of lower limbs LST is given in Table 3. The new model included ln body mass, ln leg length, ln mid-thigh circumference, ln anterior mid-thigh skinfold, ln medial calf skinfold and percent of attained PMS. Its association with lower limbs LST_DXA_ was nearly perfect (*R* = 0.95). The prediction equation was:
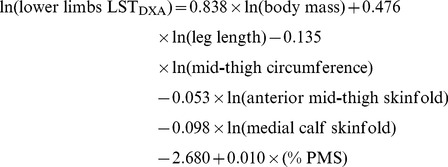
(2)


**Table 3:** Proportional multiplicative allometric regression model and internal cross-validation for the prediction of DXA-measured lower limbs lean soft tissue*.

	Predictors					Model summary			
*y*	*x*_i_	β Unstandardized	95% CI	Tolerance	VIF	*R*	SEE	Cross-validation	
								*R^2^*_PRESS_	SEE_PRESS_
ln (lower limbs LST_DXA_)	Constant	−2.680	−4.308 to −1.052						
	ln (body mass)	0.838	0.616 to 1.061	0.231	4.332				
	ln (leg length)	0.476	0.067 to 0.884	0.540	1.853				
	ln (mid-thigh circumference)	−0.135	−0.412 to 0.143	0.219	4.568				
	ln (anterior mid-thigh skinfold)	−0.053	−0.129 to 0.024	0.306	3.264				
	ln (medial calf skinfold)	−0.098	−0.176 to 0.020	0.347	2.878				
	% PMS	0.010	−0.002 to 0.021	0.480	2.084				
						0.95	0.07	0.90	0.27

Abbreviations: 95% CI, 95% confidence intervals; VIF, variance inflation factors; LST, lean soft tissue; DXA, dual x-ray absorptiometry; % PMS, percentage of attained predicted mature stature.

*ln (lower limbs LST_DXA_)  = *k*_1_ × ln (body mass) +*k*_2_ × ln (leg length) +*k*_3_ × ln (mid-thigh circumference) +*k*_4_ × ln (anterior mid-thigh skinfold) +*k*_5_ × ln (medial calf skinfold) +*a*+*b* × (% PMS) +ln *ε*.

Equation (2) (new model) was validated using PRESS statistics (Table 3). The high *R* ^2^ (0.90) and low SEE (0.27) emphasize the accuracy of the model.

**Figure 2:**
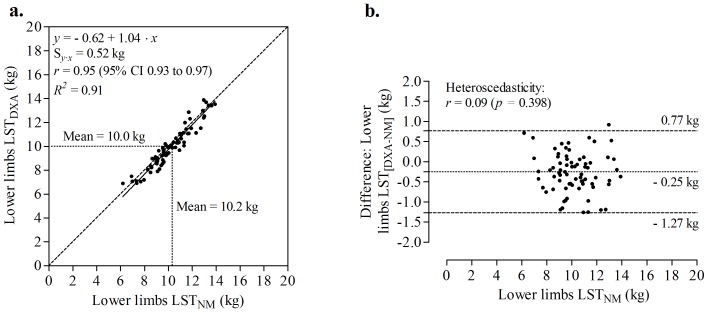
Deming regression analysis between the new anthropometric model (NM) and lower limbs lean soft tissue (LST) measured by dual-energy X-ray absorptiometry (panel a). The reference line from the equation, standard error of estimation (S*_y_*_·*x*_), correlation (*r*) and coefficient of determination (*R^2^*), are also presented. The right panel (panel b) illustrate the relation between residuals (mean differences between lower limbs LST measured by DXA and predicted by the derived equation) and lower limbs LST_NM_ (heteroscedasticity diagnostic). The dashed lines represent 95% limits of agreement (±1.96 SD).

Deming repression analysis between predicted and measured lower limbs LST showed that the new model had an *R* ^2^ = 0.91 and a S*_y·x_* of 0.52 kg (Figure 2, panel a). The intercept (−0.62, 95% CI −1.40 to 0.17) and slope (1.04, 95% CI 0.96 to 1.11) of the equation did not significantly differ (*p*>0.05) from the identity line (i.e., *y*-intercept  = 0 when *x* = 0 and slope  = 1). Therefore, the possibility of systematic or proportional bias was rejected [51]. The relation between residuals and predicted lower limbs LST values is illustrated in Figure 2 (panel b). No significant mean differences between predicted and measured values and small limits of agreement were found (bias ±1.96 SD = −0.25±1.02 kg, *p*>0.05). The measurement differences (error) relative to predicted lower limbs LST were homoscedastic [46], [47], which was confirmed by the correlation (*r* = 0.09, *p* = 0.398) between the absolute differences and the predicted lower limbs LST values [52].

## Discussion

The data detailed total body and segmental (lower limbs) LST in healthy circumpubertal boys in the context of developing and cross-validating a model that included anthropometric dimensions and a non-invasive estimate of maturity status among predictors of lower limbs LST relative to DXA as the reference method. Allometric modeling identified biological maturity status, anterior mid-thigh and medial calf skinfolds, mid-thigh circumference, leg length and body mass among significant predictors of lower limbs LST. The new model showed was valid and non-biased, and accurately predicted lower limbs LST as evident in a nearly perfect correlation between the new model and DXA, an intercept and slope that did not differ from the identity line, small limits of agreement, and errors independent of the magnitude of the values. The equation also had a high *R* ^2^_PRESS_ and low SEE_PRESS_. To our knowledge, this is the first study that obtained and cross-validated an equation to estimate lower limbs LST based on anthropometry and estimated maturity status in circumpubertal boys.

Appendicular skeletal muscle mass can be obtained from DXA [25], [53]–[55] by assuming that all non-bone and non-fat tissue is muscle mass [19]. This assumption is likely to be valid in the legs [56], [57] and is most likely to be valid at the regions between the joints such as the mid-thigh and the calf, where the amount of tendons and cartilage is small [20]. Nearly perfect correlations (*r* = 0.98–0.99) between appendicular LST assessed by DXA and skeletal muscle mass determined by MRI were noted in a multi-ethnic adolescents and children, consistent with these assumptions [58]. Total body LST of the present sample of boys 11.0±0.5 years was less (29.0±4.2 kg) than that of young boys 13.7±3.0 years (38.1±12.7 kg) [59], reflecting age and body size variation (stature: 144.8±6.4 kg *vs.* 158.1±17.7 cm; body mass: 40.2±9.0 kg *vs.* 50.2±17.4 kg, respectively).

DXA measurements of LST, body fat and bone mineral content were made independently, each with potential measurement error. In the present sample, the difference between whole body DXA mass and scale mass was 0.4 kg (0.9%). Although hydration was not controlled, minimal effects are expected since it has been shown that a subtraction or addition to the Reference Man of 1 kg in extracellular fluid would result in roughly a 0.6% over- or underestimation to total body fat mass using DXA [60]. Errors in the lower limbs LST prediction equation may be attributed in part to the use of skinfolds as predictor variables. The use of skinfold thickness measurements to estimate human body composition is based on two assumptions [61]: the thickness of the subcutaneous adipose tissue reflects a constant proportion of total body fat, and that the sites selected for measurement represent the average thickness of the subcutaneous adipose. Neither assumption, however, has been shown to be consistently generalized [62]. Skinfolds were performed by a single experienced observer following standard procedures [29], in the present study, and intra-observer technical errors of measurement (∼0.7 mm) and coefficients of variation (3.7–4.7%) were small [26].

There is no direct measure of leg length [63]. Jones and Pearson [22] measured leg as the length from the ground to the gluteal furrow. In the present study, leg length was estimated as the difference between stature and sitting height. In the DXA protocol, the leg length was defined as the mass of tissue distal to an oblique line passing through the neck of the femur, excluding gluteal muscles.

Leg circumferences were identified as suitable predictor variables. Although the correlation between stature × *CC*_m-t_^2^ with lower limbs LST_DXA_ was relatively high (*r* = 0.72, *p*<0.001), the interaction term was not a significant predictor of lower limbs LST_NM_. On the other hand, two studies [25], [31] suggested that the square of each corrected muscle girth multiplied by stature is the anthropometric parameter that contributed most to appendicular LST and skeletal muscle mass variance. The discrepancy among results and contribution of corrected muscle circumferences and stature (components of the cylinder's dimensions of the skeletal muscle mass of the human body) may be due to the considerably larger appendicular LST of young athletes [25] and non-obese adults [31] compared to this circumpubertal sample.

The adolescent growth spurt in stature starts, on average, at about 10–11 years of age in boys and reaches peak velocity (age at peak height velocity, PHV) at about 14 years [27]. Predicted time from PHV (erroneously labeled PHV), based on sex-specific anthropometric equations [64], was a significant predictor of total body LST in boys 8–18 years of age [59]. Note, however, predicted years from PHV and in turn age at PHV in boys is dependent on CA at prediction and actual age at PHV in an independent longitudinal sample of boys followed from 8 to 18 years; predicted age at PHV also has a reduced range of variation compared to actual age at PHV [65]. Identical results have been reported for an independent longitudinal sample of girls, highlighting the limitations of the prediction protocol [66]. Given the confounding effect of CA per se on the estimate of maturity timing, it is likely the effect attributed to years from PHV is due largely to CA.

Sexual maturation, based on a five stage scale using the criteria described by Tanner [67], have been considered in studies of skeletal muscle mass in boys 5–17 years [58] and appendicular LST in adolescent athletes 14–17 years [25]. Stages of secondary sex characteristics, though valuable, have limitations analytically. They indicate stage at the time of observation and do not indicate when the stage was entered or how long the individual has been in a stage. Moreover, stages of pubic hair and genital development in boys (or pubic hair and breast stages in girls) are not equivalent [27]. The present study used a non-invasive indicator of maturity status, percentage of predicted mature (adult) stature (% PMS) attained at the time of observation. Results indicated a significant and positive contribution of individual differences in % PMS to the prediction of lower limbs LST.

Although advances in technology permit accurate assessments of segmental tissue mass in vivo, assessments are limited largely to clinical or laboratory settings and are expensive. There is an interest for methods that provide information in a safe, cost-effective and non-invasive manner, and in a non-laboratory setting. The present study offers an equation that compares favorably with those reported in the literature for similar purposes [6], [25], [26], [31], [68]. No significant mean differences between measured and predicted lower limbs LST were observed, and the robustness of the model was not compromised by multicollinearity between independent variables. Tolerance (0.22–0.54) and a variance inflation factors (1.85–4.57) were well within the normal ranges (>0.10, <10, respectively) [39]. The adopted method of cross-validation [40] confirmed the effectiveness of the model to predict lower limbs LST with a high internal validity (*R* ^2^_PRESS_ = 0.90) and low proportional errors of estimation (SEE_PRESS_ = 0.27). The PRESS method avoids data-splitting difficulties and provides similar unbiased estimates of future prediction equation performance.

It was deemed important to maintain an appropriate participant-to-variable ratio. In multiple regressions, that ratio was recommended to be higher than 5∶1 and preferably 20∶1 [69]. The present ratio was ∼13∶1. Moreover, it is established that PRESS statistics usually generates less confident estimates of an equation's potential [40]. The least products regression (Deming regression), where random errors in both dependent and independent variables are accommodated in the regression model [42], was used as an alternative to the ordinary least squares method. The new model (*R* ^2^ = 0.91 and a S*_y·x_* of 0.52 kg) did not presented systematic or proportional bias as the equation did not differ significantly from the identity line [51]. Lastly, the measurement differences (error) against the predicted lower limbs LST values were homoscedastic (*r* = 0.09, *p* = 0.398) [46], [47], allowing the use of this equation with constant error variance.

Although the current investigation has a number of strengths (e.g., objective measures, analytical procedures), it is not without limitations. First, the method for estimating maturity status was non-invasive, but the prediction equation was based on middle socio-economic class American youth of White/European ancestry in the Fels Longitudinal Study [32]. Validation of the equations in Portuguese youth is needed. Second, the study was limited to a cross-sectional sample of healthy circumpubertal Portuguese boys 10–13 years of age and prediction equations tend to be age and population specific. Additional validation studies are needed to critically validate this model in other samples (e.g., mid- and late-adolescents, youth of other ethnic groups, or specific populations with varying anthropometric characteristics).

## Conclusions

In summary, a new model derived from anthropometric variables and maturity status was proposed for assessing lower limbs LST in research dealing with circumpubertal boys. The equation was satisfactorily cross-validated at group and individual basis, ensuring its applicability to similar samples. However, it is important to note that when an equation is applied to groups the associated errors will be always smaller than those for individuals. This equation will allow accurate lower limbs-specific mass information, thus offering a valid alternative to scan participants with technologies such as DXA.
